# In Memory of Two Pioneers in the Complement Field—Sir Peter J. Lachmann, 1931–2020 & Robert B. Sim, 1951–2021

**DOI:** 10.3390/v13030431

**Published:** 2021-03-08

**Authors:** Reinhard Würzner

**Affiliations:** Institute of Hygiene and Medical Microbiology, Medical University of Innsbruck, Schöpfstraβe 41, A-6020 Innsbruck, Austria; Reinhard.Wuerzner@i-med.ac.at

With deep sadness, we have to accept that two cornerstones, not just of British immunology, but also world-famous scientists in the field of complement research, passed away within the margin of a few weeks: Peter Julius Lachmann on 26 December 2020 (Figure 1), and Robert Braidwood Sim (known to everyone as Bob) on 6 February 2021 (Figure 2). Both missed a big birthday: Peter his 90th and Bob his 70th.

Both were the bricks and mortar of two complement focused Medical Research Council (MRC) Units in England: Bob as senior staff scientist of the MRC Immunochemistry Unit in Oxford (under its brilliant directors Nobel laureate Rodney Porter and Kenneth B.M. Reid), which also housed other world leading scientists including Duncan Campbell, Alex Law and Tony Day, and Peter as director of the Cambridge-based MRC Molecular Immunopathology Unit with Mike Hobart and Dick Harrison as staff scientists. Both MRC Units were centres of excellence at national and international levels and many complementologists had the opportunity to visit these places as invited seminar speakers, PhD students, post-docs, or visiting scientists, e.g., on sabbatical. Both institutions promoted complement research with pioneering contributions to the field and became safe havens and melting points fostering collaborations on a world-wide stage. Due to the age gap, Peter acted as external examiner for Bob’s Oxford D.Phil in 1976.

Peter and Bob were very different personalities, with Peter being an excellent scholar gifted with a very fast working analytical mind and an outstanding memory and the ability to brighten up any audience with his sharp and humorous comments, while Bob was a silent and very forgiving thinker who found kind words for every student who lost his track in a seminar presentation or scientific discussion and provided a dignified escape route for those who had become bogged down in a difficult point of conjecture. 

How different they were as personalities and how similar they were in advancing our field. They worked on different proteins in the complement system and, thus, there are only two publications in PubMed where both of them were co-authors on the same paper (both with senior author Wilhelm Schwaeble). Both have made seminal contributions especially related to complement and both were awarded with the gold medal for lifetime achievements by the European Complement Network, Peter at the very first occasion in 1997, and Bob in 2013. Peter was knighted and Bob received an honorary doctorate by the University of the Republic of Uruguay, Montevideo.

Peter was born in Berlin and his family moved in time to the UK. He was a medic by training and in the early days analysed conglutinin, leading to the identification and characterisation of conglutinogen activating factor, now known as Factor I, which was also the major component of his last papers. Other subjects were the dysregulation of C3 activation in disease and the “C3 tickover hypothesis,” a seminal finding explaining the role of the important alternative pathway. He was also a pioneer of the terminal cascade, elucidating the reactive lysis mechanism with Ron Thompson in his early days and the discovery and description of CD59 decades later. He was biological secretary of the Royal Society and clearly had many other scientific fields of engagement, bee keeping among these.

Bob, a Scotsman, was a biochemist by training and graduated top of the year in 1973, coming with a B.Sc. from Edinburgh to Oxford to study for a D.Phil. He remained in Oxford throughout his career apart from a two-year fellowship to work in Grenoble with Maurice Colomb and Gerard Arlaud. His main interests were the structure-functions relationships of complement proteins and their interaction with viruses, bacteria, or parasites. His favourite molecule was clearly factor H, for which he and his group provided the full amino acid sequence, the assignment of the gene to chromosome 1q, characterisation of truncated forms, polymorphisms, and local synthesis. Of special note was the strong collaboration with researchers from Uruguay on the characterisation of the role of factor H in the immune evasion of echinococcus. Bob was also very interested in the myriad relationships between the various homeostatic mechanisms in blood plasma.

At work and before his official retirement (he never retired in practice), Peter was more the senior administrator and supervisor, but never tired of disseminating extremely useful ideas when you met him in his office as his PhD student. One of these star hours is detailed elsewhere (https://pubmed.ncbi.nlm.nih.gov/33573029/ (accessed on 29 January 2021)). However, when he fished an antique (and not to be used by anybody else apparatus from the uppermost shelf), once a month or so, everybody was smiling and everybody was taking cover, except for his brave technician, Rodney Oldroyd, who performed these experiments with him. Later, with his lab at the Vet School, northwest of Cambridge and very near to his house, he was freed from too much administrative work and really went back to bench again, together with Barbara Fernie and David Seilly, until the last day.

Bob, in contrast, never really left the bench. Being only separated by a usually open door, he was always at the centre and heartbeat of his research group. When a visiting scientist arrived, he would not tell her/him to read the method in this or that book, but he would sit next to them in the lab and with his characteristic left handwriting he would hand out an individualized handwritten protocol, which usually started with “Make up 15 mL of broth and add …”. I still have these unique personal protocols. Wilhelm Schwaeble shares this memory: Bob standing at the chest freezer of his lab with a sheet of paper in his hand providing anyone that he deemed worthy with the most valuable samples and preparations, like Father Christmas readily fetching the nicest and most delicate presents out of his huge bag.

Both have had strong women at their side, Dr. Sylvia Lachmann and Prof. Edith Sim, both with their own research fields and both likely to be their spouses’ most astute advisors. Our thoughts are with their families, but especially with Sylvia and Edith.

Peter and Bob, you will be vividly remembered and greatly missed.

Reinhard Würzner
Guest Section Editor
President of the European Complement Network (ECN)
Visiting scientist in Oxford with Bob Sim (March–May 1990)
PhD student in Cambridge with Peter Lachmann (1990–1993)

## Figures and Tables

**Figure 1:**
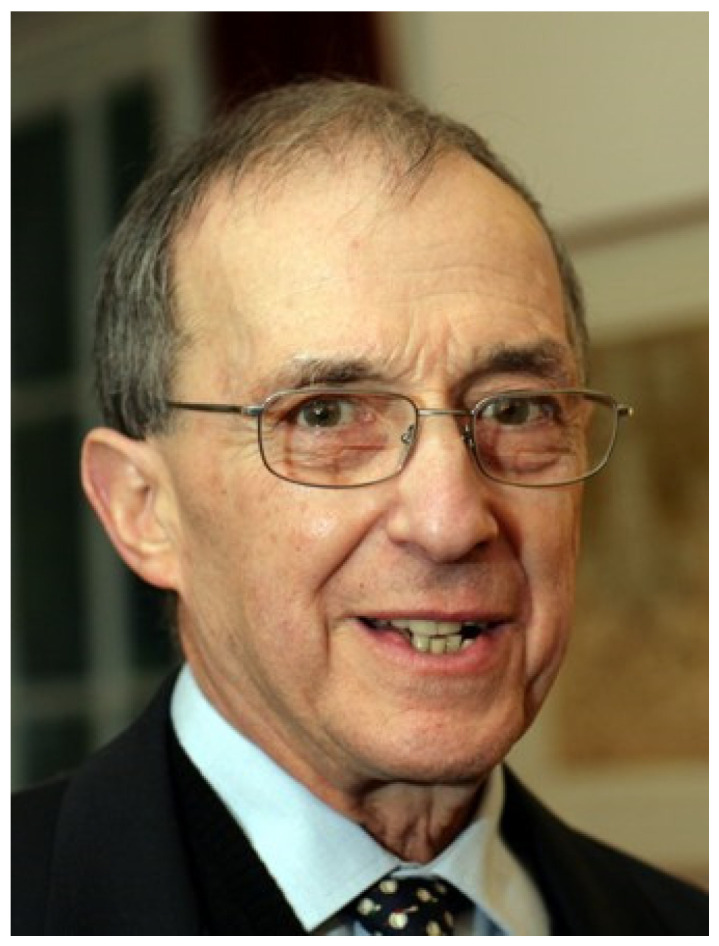
Professor Sir Peter J. Lachmann, 1931–2020 (Courtesy of Christ’s College, Cambridge).

**Figure 2:**
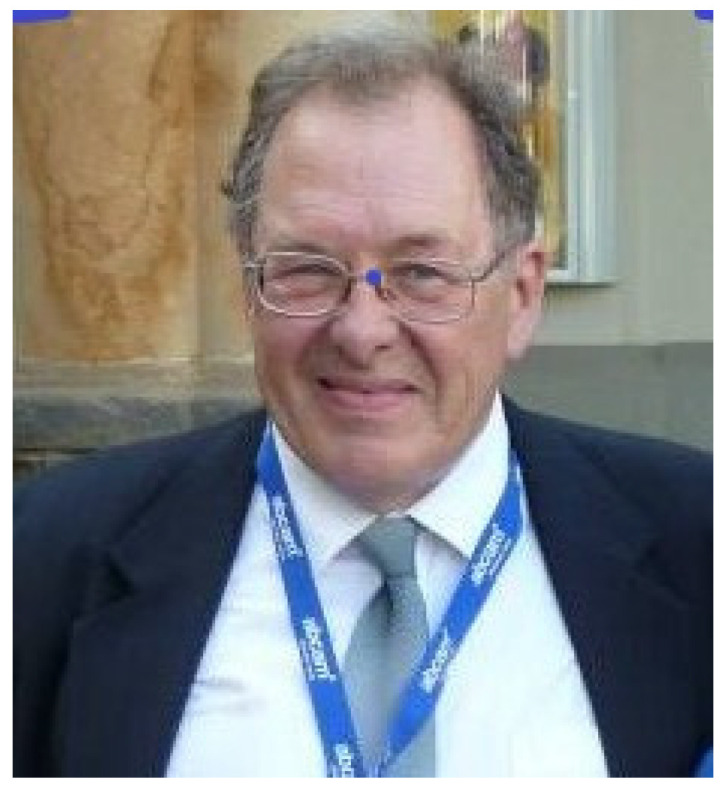
Robert B. Sim, 1951–2021 (Courtesy of Wilhelm Schwaeble, Cambridge)

